# Gestational diabetes mellitus remains the risk factor for neonatal adverse outcomes in multiparous women

**DOI:** 10.3389/fped.2025.1573470

**Published:** 2025-06-13

**Authors:** Yuxin Xiang, Siqi Zhang, Yang Li, Wenbin Dong, Qingqing Luo, Xiaoping Lei

**Affiliations:** ^1^ Division of Neonatology, Department of Pediatrics, The Affiliated Hospital of Southwest Medical University, Luzhou, China; ^2^ School of Pediatrics, Southwest Medical University, Luzhou, China; ^3^ Department of Obstetrics, The Affiliated Hospital of Southwest Medical University, Luzhou, China; ^4^ Sichuan Clinical Research Center for Birth Defects, The Affiliated Hospital of Southwest Medical University, Luzhou, China

**Keywords:** gestational diabetes mellitus, the universal two-child policy, multiparous women, adverse neonatal outcomes, long-term implications

## Abstract

**Purpose:**

Following China's universal two-child policy, the number of multiparous women increased by 90 million, coinciding with a rise in gestational diabetes mellitus (GDM). Previous studies have indicated that GDM can be effectively managed through antenatal care and lifestyle interventions. This study aims to explore whether GDM still remains a risk factor for adverse neonatal outcomes among multiparous women after the implementation of the universal two-child policy and the enhancement of antenatal care in China.

**Method:**

A total of 7,496 multiparous women were categorized into four groups: those without any complications, those with GDM only, those with non-GDM complications, and those with both GDM and non-GDM complications. Logistic regression models were employed to calculate the adjusted odds ratio (aOR) and its 95% confidence interval (CI) for each outcome. Stratified analysis (based on maternal age) and sensitivity analysis (restricted to multiparas with GDM and/or hypertensive disorders in pregnancy) were carried out to evaluate the robustness of the results.

**Results:**

Compared to infants born to multiparous women with GDM alone, infants born to multiparas without any complications had lower risks of preterm birth (PTB) (aOR 0.57, 95% CI 0.46–0.70), macrosomia (aOR 0.60, 95% CI 0.43–0.83), large for gestational age (aOR 0.53, 95% CI 0.44–0.61). When considering multiparous women with GDM with non-GDM complications, the offspring had higher risks of PTB (aOR 1.98, 95% CI 1.33–2.96), LBW (aOR 2.49, 95% CI 1.54–4.01), and small for gestational age (aOR 4.82, 95% CI 2.41–9.65).

**Conclusion:**

Despite advancements in China's prenatal care system following the two-child policy, GDM persists as a modifiable, high-impact risk factor for neonatal adverse outcomes in multiparous women. Crucially, the synergistic effects of GDM with other pregnancy complications amplify these risks, necessitating early screening (e.g., first-trimester glucose profiling), intensified glycemic management protocols, and family-centered interventions tailored to China's unique sociodemographic landscape.

## 1 Introduction

Gestational diabetes mellitus (GDM), defined as pregnancy-onset carbohydrate intolerance, is associated with heightened risks of adverse maternal-fetal outcomes and represents a significant global health challenge (1). Globally, GDM prevalence ranges from 10.4% to 25.0%, affecting approximately 1 in 7 live births (2–4). The pathogenesis of GDM primarily stems from β-cell dysfunction. Driven by accelerated apoptosis and impaired insulin production, this dysfunction fails to counteract insulin resistance, ultimately leading to hyperglycemia (5). Notably, emerging evidence highlights that these metabolic derangements are further modulated by epigenetic mechanisms, such as DNA methylation of key metabolic genes, histone modifications, and disrupted function of non-coding RNAs (ncRNAs) including microRNAs (miRNAs) (6). These epigenetic changes not only exacerbate maternal insulin resistance but also “program” long-term metabolic risks in offspring, such as β-cell dysfunction and increased susceptibility to type 2 diabetes. These metabolic derangements directly give rise to adverse clinical outcomes: GDM exerts profound clinical consequences by independently increasing risks of neonatal hyperglycemia, macrosomia, stillbirth, and intrauterine death (1, 4, 7, 8). In the long term, GDM elevates the risk of type 2 diabetes mellitus, metabolic syndrome, obesity, and cardiovascular disease in the offspring (9).

Since China introduced the universal two-child policy in 2015, profound demographic changes have emerged (10). Official data show this policy shift has led to an estimated increase of at least 90 million multiparous women, with a significant portion aged over 35 (11, 12). Consequently, certain pregnancy complications—most notably GDM—have seen a marked rise (13–15). In 2019, mainland China reported a GDM prevalence of 14.8%, with some regions exceeding 20.9% (16, 17). Zhejiang Province has witnessed a 42% post-policy GDM rise (2016–2018), straining healthcare systems (18). This significant increase in GDM prevalence poses a substantial burden on public health systems.

Despite these challenges, it is important to note that several studies indicate GDM can be effectively managed through antenatal care and lifestyle interventions (19–21). There even exists a perspective that, when compared with other pregnancy complications, GDM may no longer be regarded as a high-risk condition (22). Following the implementation of the universal two-child policy, evidence indicates that women opting for a second pregnancy tend to belong to higher socioeconomic status (SES) groups (22). Over the past decade, China has achieved significant improvements in antenatal care and neonatal care practices (12). However, despite these advancements, research remains limited on whether GDM continues to serve as a critical risk factor for neonatal outcomes in subsequent pregnancies. Despite the growing burden of GDM in China, whether it remains a critical risk factor for adverse neonatal outcomes in multiparous women post-policy implementation remains unclear. This study addresses this knowledge gap by examining associations between GDM and neonatal outcomes in a demographically representative cohort. Since the policy shift in 2015, many multiparous women were among the first to embrace the two-child policy, constituting a socially representative cohort during this transitional period. This policy transition presents a unique opportunity to investigate associations between GDM, non-GDM pregnancy complications, and adverse neonatal outcomes in multiparous women.

## 2 Methods

### 2.1 Participants

The data were obtained from a registered cohort: a hospital-based cohort from the Maternity and Child Registration System supported by the Health Commission of Luzhou City, spanning December 1, 2015, to December 1, 2020. This cohort was established to track and supervise pregnant multiparous women delivering in all hospitals within Luzhou District and their fetuses/newborns. The project and protocol underwent rigorous review and were granted approval by the Ethics Committee of The Affiliated Hospital of Southwest Medical University (No. KY2021264).

**Figure 1:**
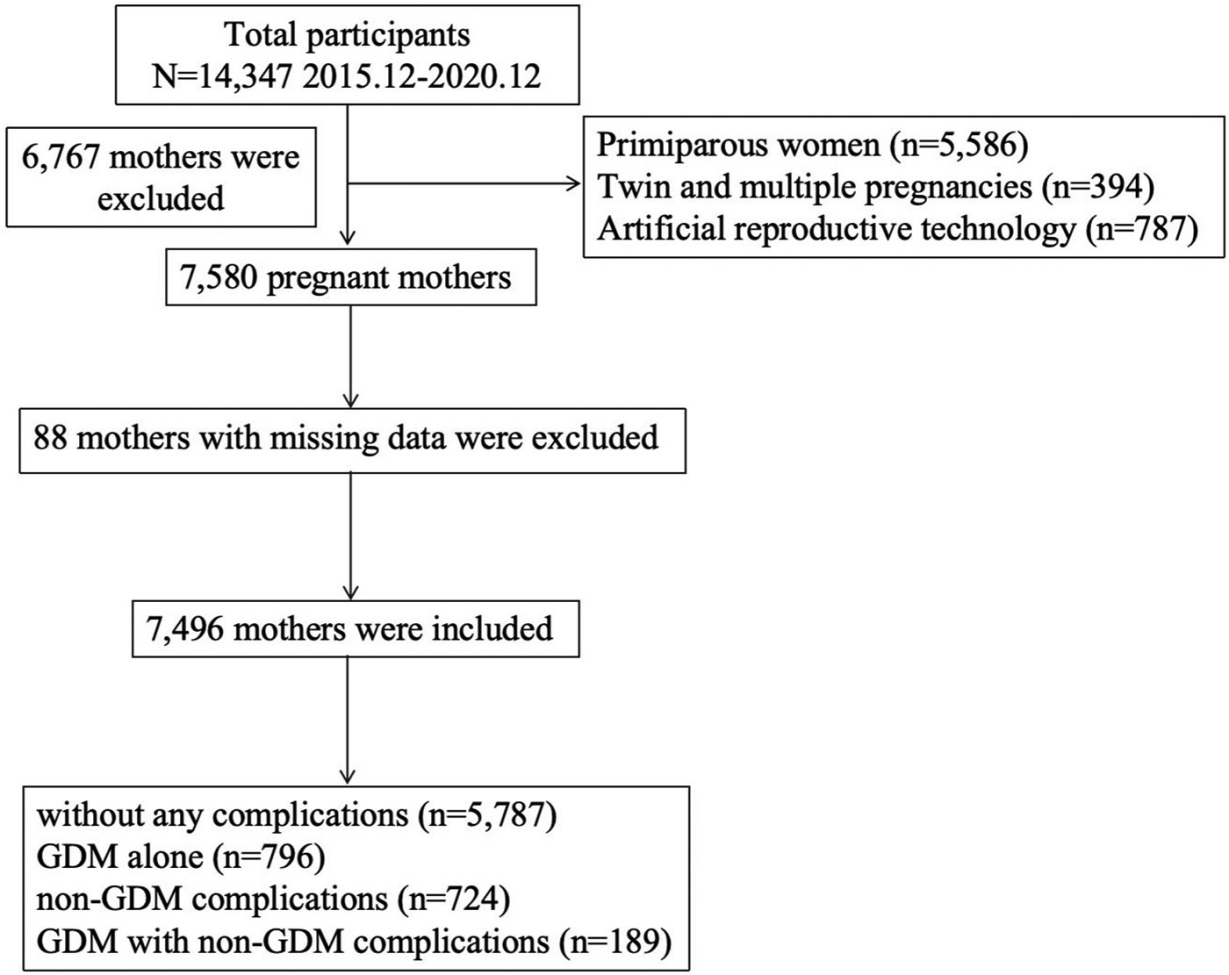
The flowchart of the whole process.

During the study duration, a total of 14,347 pregnant women were followed. Initial screening excluded 5,586 primiparous women. Subsequently, pregnancies involving twins or multiples (*n* = 394) and those utilizing artificial reproductive technology (*n* = 787) were also eliminated. Additionally, we excluded multiparous women with missing birth weight information for their offspring (*n* = 84). The final cohort consisted of 7,496 eligible multiparous women (Figure 1).

Among 7,496 participants, interpregnancy interval (IPI) were missing for 77 cases (1.03%, group range: 0.36%–6.42%), and body mass index (BMI) data was unavailable for 605 (8.07%, group range: 7.55%–11.6%).

### 2.2 Maternal pregnancy complications

The maternal pregnancy complications in the present study included GDM, hypertensive disorders in pregnancy (HDP), intrahepatic cholestasis of pregnancy (ICP), thyroid disease of pregnancy, and other pregnancy complications diagnosed by obstetricians. GDM was diagnosed according to American Diabetes Association (ADA) criteria: the diagnosis of GDM was made when any one of the 75 g oral glucose tolerance test (OGTT) values met or exceeded 5.1 mmol/L at 0 h, 10.0 mmol/L at 1 h, and 8.5 mmol/L at 2 h when performed between 24 and 28 gestational weeks (23) and HbA1c was used to monitor glycemic control. HDP included gestational hypertension, pre-eclampsia, eclampsia, pre-eclampsia superimposed upon chronic hypertension, and pregnancy with chronic hypertension (24). ICP was diagnosed based on Obstetrics Group of Obstetrics and Gynecology Branch of Chinese Medical Association with a new one-set pruritus with total bile acids level >10 µmol/L (25). Thyroid diseases during pregnancy were diagnosed based on the guideline of American College of Obstetricians and Gynecologists (ACOG) including at least one of hyperthyroidism, subclinical hyperthyroidism, hypothyroidism, or subclinical hypothyroidism during pregnancy (25, 26).

Multiparous women were stratified into four groups based on pregnancy complications: (1) no complications (*n* = 5,787), (2) GDM alone (*n* = 796), (3) non-GDM complications (*n* = 724), and (4) GDM with non-GDM complications (*n* = 189).

### 2.3 Adverse neonatal outcomes

The neonatal outcomes included preterm birth (PTB, gestational age <37 weeks), low birth weight (LBW, birth weight <2,500 g), macrosomia (birth weight ≥ 4,000 g), small for gestational age (SGA, defined as a birth weight < the 10th percentile for gestational age) (27), large for gestational age (LGA, defined as a birth weight > the 90th percentile for gestational age) (28), low Apgar score (including 1- or 5-min Apgar score <7), and severe adverse neonatal outcomes (at least one of stillbirth, resuscitation failure in the delivery room, or admission to NICU). Notably, isolated low Apgar score (without subsequent organ dysfunction) show insufficient evidence for association with long-term neurodevelopmental impairment and cannot reliably predict individual morbidity or mortality risks (29). Severe adverse outcomes may be associated with long-term prognosis, as they often indicate more serious neonatal complications.

### 2.4 Covariates

Demographic characteristics and diagnostic information were extracted from the hospital information system, including maternal age, gravidity, parity, IPI (months), BMI (kg/m²), abortion history (yes/no), current delivery mode (vaginal/cesarean), and neonatal gestational age and birth weight.

### 2.5 Statistical analysis

Continuous variables with normal distribution were presented as means ± standard deviations (SD) and compared using analysis of variance (ANOVA). Non-normally distributed variables were described using median (interquartile range, IQR) and compared via the Wilcoxon test. Categorical variables were presented as frequencies (percentages) and tested using the chi-squared test (*χ*^2^), while ordered variables were analyzed using the Cochran-Mantel-Haenszel chi-squared test (*χ*^2^).

To determine whether GDM remained a risk factor for neonatal adverse outcomes—with lower risks than other non-GDM complications—multiparous women with GDM alone were designated as the reference group. Logistic regression models were used to adjust for potential confounders, calculating odds ratios (ORs) and 95% confidence intervals (CIs). The adjusted model controlled for: gravidity (2, 3, >3), parity (2, >2), BMI, abortion history (yes/no), delivery mode (vaginal/cesarean), IPI, gestational age, birth weight, and maternal age. Maternal age was a strong predictor for GDM and other pregnancy complications; stratified analyses were conducted in different maternal age groups (<35 and ≥35 years).

Because of the low incidence of ICP, thyroid disease of pregnancy, and other complications, we conducted a sensitivity analysis in a population that only included multiparous women with GDM or HDP. The neonatal outcomes were compared among multiparous women without any complications (*n* = 5,787), with GDM alone (*n* = 796), with HDP alone (*n* = 391), and with both GDM and HDP (*n* = 105) to test the robustness of the results.

The statistical analyses were performed using SAS software 9.4 (SAS Institute, Inc., Cary, NC, USA). All *p*-values are two-tailed, and a *p*-value <0.05 was considered significant.

## 3 Results

Table 1 reveals differences in maternal and neonatal characteristics among the four groups. Significant differences were observed in maternal age, gravidity, IPI, BMI, abortion history, mode of the current delivery, birth weight, and gestational age (all *p* < 0.05).

**Table 1:** Maternal and neonatal characteristics among pregnant multiparous women with different pregnancy complications.

Maternal and neonatal characteristics	All subjects	Without any complications	GDM alone	Non-GDM complication^a^	GDM with non-GDM complications	*P*
	*N* = 7,496	*n* = 5,787	*n* = 796	*n* = 724	*n* = 189	
Maternal age [year, median (IQR)]	31 (7)	31 (7)	33 (7)	33 (8)	35 (5)	<0.01
<35	5,350 (71.37)	4,312 (74.51)	495 (62.19)	450 (62.15)	93 (49.21)	<0.01
≥35	2,146 (28.63)	1,475 (25.49)	301 (37.81)	274 (37.85)	96 (50.79)	
Gravidity						<0.05
2	1,860 (24.81)	1,482 (25.61)	187 (23.49)	158 (21.81)	33 (17.46)	
3	2,131 (28.43)	1,652 (28.55)	219 (27.52)	209 (28.87)	51 (26.98)	
>3	3,505 (46.76)	2,653 (45.84)	390 (48.99)	357 (49.32)	105 (55.56)	
Parity						0.07
2	5,906 (78.79)	4,565 (78.88)	646 (81.16)	554 (76.52)	141 (74.60)	
>2	1,590 (21.21)	1,222 (21.12)	150 (18.84)	170 (23.48)	48 (25.40)	
Interpregnancy interval [month, median (IQR)]	62 (72)	50 (61)	74 (74)	72 (82)	87 (84)	<0.01
<24	1,220 (16.27)	1,039 (17.95)	82 (10.30)	83 (11.47)	16 (8.47)	<0.01
24–60	2,479 (33.07)	2,035 (35.16)	198 (24.87)	205 (28.31)	41 (21.69)	
>60	3,720 (49.63)	2,693 (46.53)	465 (58.41)	436 (60.22)	126 (66.67)	
Missing	77 (1.03)	20 (0.36)	51 (6.42)	0	6 (3.17)	
Body mass index [kg/m^2^, median (IQR)]	26.70 (4.30)	26.60 (4.20)	27.30 (4.90)	27.30 (5.40)	29.55 (6.00)	<0.01
<25	1,876 (25.03)	1,517 (26.21)	177 (22.24)	157 (21.69)	25 (13.23)	<0.01
≥25	5,015 (66.90)	3,833 (66.24)	556 (69.85)	483 (66.71)	143 (75.66)	
Missing	605 (8.07)	437 (7.55)	63 (7.91)	84 (11.60)	21 (11.11)	
Abortion history						<0.01
Yes	5,182 (69.13)	3,945 (68.17)	579 (72.74)	516 (71.27)	142 (75.13)	
No	2,314 (30.87)	1,842 (31.83)	217 (27.26)	208 (28.73)	47 (24.87)	
Mode of the current delivery						<0.01
Vaginal	2,438 (32.52)	1,943 (33.58)	230 (28.89)	226 (31.22)	39 (20.63)	
Cesarean	5,058 (67.48)	3,844 (66.42)	566 (71.11)	498 (68.78)	150 (79.37)	
Birth weight [g, median (IQR)]	3,200 (660)	3,210 (630)	3,280 (713)	3,035 (1,010)	3,140 (930)	<0.01
Gestational age [week, median (IQR)]	38 (2)	38 (1)	38 (2)	38 (3)	38 (3)	<0.01

^a^ Non-GDM complications included hypertensive disorders in pregnancy, intrahepatic cholestasis of pregnancy, thyroid disease of pregnancy and other pregnancy complications diagnosed by obstetrician.

Compared to infants born to multiparous women with GDM alone, infants born to mothers without any complications had lower risks of PTB (aOR 0.57, 95% CI 0.46–0.70), macrosomia (aOR 0.60, 95% CI 0.43–0.83), LGA (aOR 0.53, 95% CI 0.44–0.61). For LBW (aOR 0.77, 95% CI: 0.58–1.00) and severe adverse neonatal outcomes (aOR 0.72, 95% CI 0.53–1.00), results should be interpreted as inconclusive evidence of an effect, though potentially indicative of a protective trend requiring validation. No differences in SGA and low Apgar score were observed between the two groups.

In contrast, multiparous women with non-GDM complications had higher risks of PTB (aOR 1.38, 95% CI 1.06–1.81), LBW (aOR 2.64, 95% CI 1.93–3.61), and SGA (aOR 7.16, 95% CI 4.28–11.99) in their offspring, but lower risks of macrosomia (aOR 0.49, 95% CI 0.30–0.80) and LGA (aOR 0.39, 95% CI 0.28–0.53). Multiparous women with both GDM and non-GDM complications had elevated risks of PTB (aOR 1.98, 95% CI: 1.33–2.96), LBW (aOR 2.49, 95% CI: 1.54–4.01), and SGA (aOR 4.82, 95% CI: 2.41–9.65). However, neither non-GDM complications nor combined GDM with non-GDM complications were associated with low Apgar score or severe neonatal outcomes compared to GDM alone (Table 2).

**Table 2:** The different associations between gestational diabetes mellitus (GDM) and non-GDM complications with neonatal adverse outcomes.

Neonatal outcomes	Without any complications	GDM alone	Non-GDM complications^a^	GDM with non-GDM complications
	*n* = 5,787	*n* = 796	*n* = 724	*n* = 189
Preterm birth^b^				
*n*/*N* (%)	927 (16.02)	169 (21.23)	219 (30.25)	62 (32.80)
OR	0.70 (0.59–0.85)	1	1.60 (1.27–2.03)	1.81 (1.27–2.56)
aOR	0.57 (0.46–0.70)	1	1.38 (1.06–1.81)	1.98 (1.33–2.96)
Low birth weight^b^				
*n*/*N* (%)	674 (11.65)	97 (12.19)	205 (28.31)	42 (22.22)
OR	0.95 (0.75–1.19)	1	2.84 (2.18–3.71)	2.05 (1.37–3.08)
aOR	0.77 (0.58–1.00)	1	2.64 (1.93–3.61)	2.49 (1.54–4.01)
Macrosomia^b^				
*n*/*N* (%)	248 (4.29)	56 (7.04)	29 (4.01)	23 (12.17)
OR	0.59 (0.43–0.79)	1	0.55 (0.34–0.87)	1.83 (1.09–3.06)
aOR	0.60 (0.43–0.83)	1	0.49 (0.30–0.80)	1.24 (0.69–2.20)
Small for gestational age^b^				
*n*/*N* (%)	267 (4.61)	22 (2.76)	149 (20.58)	27 (14.29)
OR	1.70 (1.09–2.64)	1	9.11 (5.75–14.44)	5.86 (3.25–10.55)
aOR	1.36 (0.83–2.23)	1	7.16 (4.28–11.99)	4.82 (2.41–9.65)
Large for gestational age^b^				
*n*/*N* (%)	775 (13.39)	184 (23.11)	86 (11.87)	51 (26.98)
OR	0.51 (0.42–0.61)	1	0.44 (0.33–0.59)	1.22 (0.85–1.75)
aOR	0.53 (0.44–0.66)	1	0.39 (0.28–0.53)	0.91 (0.60–1.36)
Low Apgar score^c^				
*n*/*N* (%)	216 (3.73)	31 (3.89)	66 (9.12)	17 (8.99)
OR	0.95 (0.65–1.40)	1	2.47 (1.59–3.84)	2.43 (1.32–4.50)
aOR	0.76 (0.42–1.37)	1	1.03 (0.51–2.10)	1.20 (0.44–3.25)
Severe adverse neonatal outcomes^c^				
*n*/*N* (%)	535 (9.24)	94 (11.81)	141 (19.48)	41 (21.69)
OR	0.76 (0.60–0.96)	1	1.80 (1.36–2.39)	2.06 (1.37–3.11)
aOR	0.72 (0.53–1.00)	1	0.93 (0.61–1.43)	1.13 (0.60–2.12)

Low Apgar score: 1- or 5-min Apgar score <7. Severe adverse neonatal outcomes: including at least one of stillbirth, resuscitation failure in delivery room, or admission to NICU.

^a^ Non-GDM complications included hypertensive disorders in pregnancy, intrahepatic cholestasis of pregnancy, thyroid disease of pregnancy and other pregnancy complications diagnosed by obstetrician.

^b^ Adjusted gravidity (2, 3, >3), parity (2, >2), body mass index, abortion history (yes/no), interpregnancy interval and maternal age.

^c^ Adjusted gravidity (2, 3, >3), parity (2, >2), body mass index, abortion history (yes/no), interpregnancy interval, maternal age, mode of the current delivery (vaginal/cesarean), gestational age, and birth weight.

**Figure 2:**
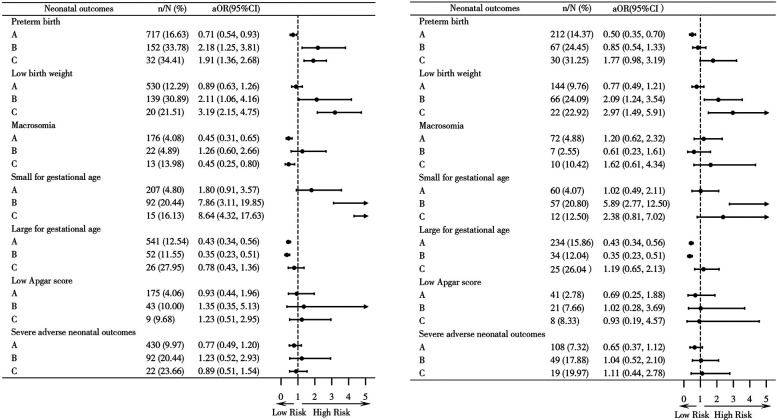
The different associations between gestational diabetes mellitus (GDM) and non-GDM complications with neonatal adverse outcomes, stratified by maternal age. **(a)** Maternal age <35 years; **(b)** Maternal age ≥35 years; A, without any complications; B, non-GDM complications; C, GDM with non-GDM complications. Preterm birth, low birth weight, macrosomia, small for gestational age, large for gestational age: adjusted gravidity (2, 3, >3), parity (2, >2), body mass index, abortion history (yes/no), interpregnancy interval. Low Apgar score, severe adverse neonatal outcomes: adjusted gravidity (2, 3, >3), parity (2, >2), body mass index, abortion history (yes/no), mode of the current delivery (vaginal/cesarean), interpregnancy interval, gestational age, birth weight.

When stratified by maternal age (<35 and ≥35 years), similar trends were observed, although statistical significance was not reached in some subgroups (Figure 2 and Supplementary Table S1). In a sensitivity analysis limited to GDM and HDP (excluding rare complications), consistent OR trends for outcomes were observed across groups (Supplementary Table S2).

## 4 Discussion

In the present study, it has been observed that GDM continues to pose a significant risk for adverse neonatal outcomes in multiparous women. Furthermore, when GDM is combined with other pregnancy complications, the risks of some adverse neonatal outcomes will increase.

Over the past decade, numerous studies have shown that the association between GDM and adverse neonatal outcomes has become less pronounced with the adoption of modern antenatal care practices (18–20). A recent investigation has emphasized that early screening for GDM, coupled with appropriate management strategies for women at risk, can lead to improved neonatal outcomes, including a reduction in emergency cesarean sections and cases of macrosomia (aOR 0.62, 95% CI 0.43–0.91) (30). After the implementation of the Chinese universal two-child policy, the government has rolled out measures to strengthen prenatal care, including the establishment of pregnancy risk assessment and management systems (31). Particularly, multiparous women will be categorized into high-risk levels according to maternal pregnancy risk assessment and management norms in China, if they meet any of the following: (1) advanced maternal age; (2) severe pregnancy complications/comorbidities; (3) a pre-pregnancy BMI ≥ 28 kg/m² (32). Obstetricians will increase follow-up visits and closely monitor glycemic control for high-risk cases, while medical institutions will maintain detailed records to ensure comprehensive care. Concurrently, multiparous women—often willing to have a second child—exhibit higher social and SES and stronger family support (22). Their enhanced SES and familial resources enable better pregnancy preparedness, including heightened attention to maternal health. Notably, National Bureau of Statistics data show that by 2016, following the universal two-child policy, the nationwide proportion of second-born children and pregnant women of advanced maternal age (≥35 years) had increased (10). Despite this demographic shift toward higher-risk pregnancies, maternal mortality continued to decline with urban and rural maternal mortality rates decreasing by 47.8% and 33.9%, respectively, from 2010 to 2018 and indicators such as antenatal examination rates and postnatal home visit rates improved to varying degrees (33). Antenatal care utilization rose significantly—evidenced by an increase in the maternal system management rate from 90.0% in 2014 to 92.9% in 2022 (34). These improvements may mitigate some adverse effects of GDM on newborns. However, our findings indicate that GDM remains independently associated with elevated incidence of PTB, LBW, macrosomia, LGA, and severe neonatal complications.

Globally, the prevalence of fetal macrosomia among mothers with GDM is estimated to be between 10% and 30% (35). The mechanisms and pathways explaining the relationship between maternal hyperglycemia and neonatal birth weight remain poorly understood (36). A leading hypothesis posits that maternal hyperglycemia, coupled with hyperinsulinemia, promotes fetal fat and protein deposition, driving excessive fetal growth (19, 37). Notably, in our study cohort, GDM screening was typically performed at 24–28 weeks' gestation—a period considered relatively late in gestation, as critical changes in fetal growth rates have already occurred (38, 39). Actually, GDM develops when preexisting β-cell dysfunction overlaps with pregnancy-induced insulin resistance (40). These combined defects not only drive hyperglycemia but also elevate lifelong diabetes risk (41). Consequently, despite enhanced antenatal care, certain neonatal complications, such as PTB, macrosomia and LGA remain challenging to prevent.

Furthermore, GDM is associated with long-term metabolic sequelae in offspring, including elevated risks of type 2 diabetes mellitus (9). Although the pathogenic mechanisms underlying these abnormal metabolic characteristics remain unclear, studies suggest that maternal hyperglycaemia may induce changes in DNA methylation and microRNA (miRNA) content in fetal blood, skeletal muscle and adipose tissue (4). Actually, this study is focused on the short-term effects of GDM in multiparous women on neonatal outcomes. Future longitudinal research should prospectively assess metabolic parameters in offspring and maternal glycometabolic status. Meanwhile, women with a history of GDM who exhibit significant risk factors—including advanced maternal age in subsequent pregnancies, elevated pre-pregnancy BMI, higher 1- and 2-hour OGTT glucose levels during prior pregnancy, and prior macrosomia delivery—face a heightened risk of recurrence (42, 43). Moreover, multiparous women had a higher recurrence rate compared with primiparous women (73% and 40%, respectively; *P* < 0.01) (42). Notably, pregnancy outcomes and complications associated with recurrent GDM are often more severe than those observed in first-time GDM cases (42). Therefore, healthcare professionals must remain vigilant in recognizing and addressing the potential short- and long-term implications of GDM on both mothers and children.

It is also noteworthy that the coexistence of GDM with other pregnancy complications significantly escalates the risks of adverse neonatal outcomes. This is particularly true when GDM co-occurs with HDP, as it leads to notably higher rates of adverse neonatal outcomes such as LBW, low Apgar scores, SGA (44). Furthermore, the concurrent presence of GDM and other complications is associated with an elevated risk of long-term morbidities, including type 2 diabetes, hypertension, and cardiovascular diseases (45). Therefore, there is a critical need to emphasize the importance of enhanced monitoring and multidisciplinary management for pregnant women with comorbid GDM and other pregnancy complications to optimize maternal and fetal outcomes.

For obstetricians, it is crucial to identify women with GDM and implement effective strategies such as lifestyle modifications, insulin therapy, continuous glucose monitoring, and oral antidiabetic medications. These interventions have been proven beneficial for both mothers and fetuses. However, clinicians must remain cautious: even though the country has strengthened its control over GDM and multiparous women have a higher socioeconomic level, the short-term adverse effects of diabetes on newborns still cannot be completely avoided.

Despite substantial advancements in national GDM management frameworks, urban-rural disparities remain pronounced. Standardized care implementation lags significantly in resource-limited regions, with studies consistently reporting screening rates below 50% in some rural areas (19). Therefore, China should leverage community hospitals to deliver timely and accessible care for pregnant women while providing antenatal education on GDM self-management. Obstetricians must also strengthen education for pregnant women about the importance of proactive GDM self-care. Moreover, the long-term impacts of GDM on both mothers and infants should not be overlooked. Ongoing surveillance and multidisciplinary follow-up are essential to mitigate risks of delayed complications, such as type 2 diabetes and cardiovascular disease, in affected individuals.

The study conducted during the special period has provided valuable insights into the healthcare of mothers and newborns. The large sample size strengthens the internal validity of our findings. However, we acknowledge some limitations. First, comprehensive data on multiparous women—such as educational background, SES, detailed GDM treatment protocols, and exact follow-up frequencies—were lacking. Despite China's efforts to improve maternal and neonatal healthcare outcomes through measures implemented after the universal two-child policy, we were unable to determine if each individual multiparous women received standardized antenatal care in our study. This limitation hindered our ability to fully elucidate how enhanced antenatal care mitigated GDM-related adverse effects. Second, the data collected may not be entirely representative, although it was authentic. Multicenter studies with larger cohorts are needed to validate our findings and assess their generalizability across diverse populations. At the same time, it should be specifically noted that this study did not systematically collect data on medication adherence or compliance with lifestyle interventions. This methodological limitation may introduce estimation bias, particularly an overestimation of the theoretical efficacy of interventions. Although sensitivity analyses validated the robustness of our results, future investigations must incorporate real-time adherence monitoring through integrated electronic health records (EHRs) and mobile health (mHealth) platforms to enhance precision.

## 5 Conclusion

Despite advancements in China's prenatal care system following the two-child policy, GDM persists as a modifiable, high-impact risk factor for neonatal adverse outcomes in multiparous women. Crucially, the synergistic effects of GDM with other pregnancy complications amplify these risks, necessitating early screening (e.g., first-trimester glucose profiling), intensified glycemic management protocols, and family-centered interventions tailored to China's unique sociodemographic landscape.

## Data Availability

The raw data supporting the conclusions of this article will be made available by the authors, without undue reservation.
