# Supplementary material for: Gestational diabetes mellitus remains the risk factor for neonatal adverse outcomes in multiparous women
Source: Front Pediatr. 2025 Jun 13;13:1573470. doi: 10.3389/fped.2025.1573470 (PMC12202611; doi:10.3389/fped.2025.1573470)
Supplement: Supplementary file 1 [file Table1.docx]

**Gestational Diabetes Mellitus Remains the Risk Factor for Neonatal Adverse Outcomes in Multiparous Women**

Table S1. The different associations between gestational diabetes mellitus (GDM) and non-GDM complications with neonatal adverse outcomes, stratified by maternal age.

| Neonatal outcomes | Without any complications  n=4312 | GDM alone  n=495 | Non-GDM complications  n=450 | GDM with non-GDM complications  n=93 | Without any complications  n=1475 | GDM alone  n=301 | Non-GDM complications  n=274 | GDM with non-GDM complications  n=96 |
| --- | --- | --- | --- | --- | --- | --- | --- | --- |
|  | < 35 years | | | | ≥ 35 years | | | |
| Preterm birth^a^ | |  |  |  |  |  |  |  |
| n/N (%) | 717 (16.63) | 102 (20.61) | 152 (33.78) | 32 (34.41) | 212 (14.37) | 67 (22.26) | 67 (24.45) | 30 (31.25) |
| OR | 0.76 (0.60-0.96) | 1 | 2.02 (1.25-3.26) | 1.96 (1.46-2.63) | 0.58 (0.43-0.79) | 1 | 1.13 (0.76-1.66) | 1.58 (0.95-2.64) |
| aOR | 0.71 (0.54-0.93) | 1 | 2.18 (1.25-3.81) | 1.91 (1.36-2.68) | 0.50 (0.35-0.70) | 1 | 0.85 (0.54-1.33) | 1.77 (0.98-3.19) |
| Low birth weight^a^ | |  |  |  |  |  |  |  |
| n/N (%) | 530 (12.29) | 62 (12.53) | 139 (30.89) | 20 (21.51) | 144 (9.76) | 35 (11.63) | 66 (24.09) | 22 (22.92) |
| OR | 0.97 (0.73-1.29) | 1 | 1.91 (1.09-3.35) | 3.12 (2.23-4.35) | 0.82 (0.55-1.21) | 1 | 2.41 (1.54-3.77) | 2.26 (1.25-4.08) |
| aOR | 0.89 (0.63-1.26) | 1 | 2.11 (1.06-4.16) | 3.19 (2.15-4.75) | 0.77 (0.49-1.19) | 1 | 2.09 (1.24-3.54) | 2.97 (1.49-5.91) |
| Macrosomia^a^ | |  |  |  |  |  |  |  |
| n/N (%) | 176 (4.08) | 42 (8.48) | 22 (4.89) | 13 (13.98) | 72 (4.88) | 14 (4.65) | 7 (2.55) | 10 (10.42) |
| OR | 0.45 (0.32-0.65) | 1 | 1.75 (0.90-3.41) | 0.55 (0.32-0.94) | 1.05 (0.58-1.89) | 1 | 0.53 (0.21-1.35) | 2.38 (1.02-5.55) |
| aOR | 0.45 (0.31-0.65) | 1 | 1.26 (0.60-2.66) | 0.45 (0.25-0.80) | 1.20 (0.62-2.32) | 1 | 0.61 (0.23-1.61) | 1.62 (0.61-4.34) |
| Small for gestational age^a^ | |  |  |  |  |  |  |  |
| n/N (%) | 207 (4.80) | 13 (2.63) | 92 (20.44) | 15 (16.13) | 60 (4.07) | 9 (2.99) | 57 (20.80) | 12 (12.50) |
| OR | 1.87 (1.05-3.30) | 1 | 7.13 (3.26-15.55) | 9.52 (5.24-17.30) | 1.37 (0.67-2.80) | 1 | 8.52 (4.12-17.59) | 4.63 (1.88-11.37) |
| aOR | 1.80 (0.91-3.57) | 1 | 7.86 (3.11-19.85) | 8.64 (4.23-17.63) | 1.02 (0.49-2.11) | 1 | 5.89 (2.77-12.50) | 2.38 (0.81-7.02) |
| Large for gestational age^a^ | |  |  |  |  |  |  |  |
| n/N (%) | 541 (12.54) | 121 (24.44) | 52 (11.55) | 26 (27.95) | 234 (15.86) | 63 (20.93) | 34 (12.40) | 25 (26.04) |
| OR | 0.44 (0.35-0.57) | 1 | 0.40 (0.28-15.55) | 1.19 (0.71-1.95) | 0.71 (0.52-0.97) | 1 | 0.55 (0.33-0.83) | 1.33 (0.23-0.51) |
| aOR | 0.35 (0.23-0.56) | 1 | 0.35 (0.23-0.51) | 0.78 (0.43-1.36) | 0.43 (0.34-0.56) | 1 | 0.35 (0.23-0.51) | 1.19 (0.65-2.13) |
| Low Apgar score^b^ | |  |  |  |  |  |  |  |
| n/N (%) | 175 (4.06) | 21 (4.24) | 45 (10.00) | 9 (9.68) | 41 (2.78) | 10 (3.32) | 21 (7.66) | 8 (8.33) |
| OR | 0.95 (0.60-1.51) | 1 | 2.41 (1.07-5.46) | 2.50 (1.46-4.28) | 0.83 (0.41-1.68) | 1 | 2.41 (1.11-5.22) | 2.64 (1.01-6.90) |
| aOR | 0.93 (0.44-1.96) | 1 | 1.35 (0.35-5.13) | 1.23 (0.51-2.95) | 0.69 (0.25-1.88) | 1 | 1.02 (0.28-3.69) | 0.93 (0.19-4.57) |
| Severe adverse neonatal outcomes^b^ | |  |  |  |  |  |  |  |
| n/N (%) | 430 (9.97) | 60 (12.12) | 92 (20.44) | 22 (23.66) | 108 (7.32) | 34 (11.30) | 49 (17.88) | 19 (19.97) |
| OR | 0.80 (0.60-1.07) | 1 | 2.24 (1.29-3.89) | 1.86 (1.30-2.65) | 0.62 (0.41-0.93) | 1 | 1.71 (1.06-2.74) | 1.93 (1.04-3.58) |
| aOR | 0.77 (0.49-1.20) | 1 | 1.23 (0.52-2.93) | 0.89 (0.51-1.54) | 0.65 (0.37-1.12) | 1 | 1.04 (0.52-2.10) | 1.11 (0.44-2.78) |

*Non-GDM complications included hypertensive disorders in pregnancy, intrahepatic cholestasis of pregnancy, thyroid disease of pregnancy and other pregnancy complications diagnosed by obstetrician.

Low Apgar score: 1-min or 5-min Apgar score < 7.

Severe adverse neonatal outcomes: including at least one of stillbirth, resuscitation failure in delivery room, or admission to NICU.

^a^ adjusted gravidity (2, 3, >3), parity (2, > 2), body mass index, abortion history (yes/no), interpregnancy interval.

^b^ adjusted gravidity (2, 3, >3), parity (2, > 2), body mass index, abortion history (yes/no), interpregnancy interval, and mode of the last delivery (vaginal/cesarean).

Table S2. The different associations between gestational diabetes mellitus (GDM) and hypertensive disorders in pregnancy (HDP) with neoantal adverse outcomes.

| Neonatal outcomes | Without any complications  n=5787 | GDM alone  n=796 | HDP alone  n=391 | GDM combing HDP  n=105 |
| --- | --- | --- | --- | --- |
| Preterm birth^a^ |  |  |  |  |
| n/N (%) | 929 (16.05) | 169 (21.23) | 143 (36.57) | 41 (39.04) |
| OR | 0.70 (0.59- 0.85) | 1 | 2.13 (1.63- 2.79) | 2.37 (1.55- 3.64) |
| aOR | 0.57 (0.46- 0.70) | 1 | 1.94 (1.42- 2.65) | 2.54 (1.52- 4.22) |
| Low birth weight^a^ |  |  |  |  |
| n/N (%) | 674 (11.65) | 97 (12.19) | 156 (39.90) | 30 (28.57) |
| OR | 0.95 (0.75- 1.19) | 1 | 4.78 (3.56- 6.41) | 2.88 (1.79- 4.62) |
| aOR | 0.76 (0.58- 0.99) | 1 | 5.54 (3.90- 7.88) | 3.71 (2.06- 6.70) |
| Macrosomia^a^ |  |  |  |  |
| n/N (%) | 248 (4.29) | 56 (7.04) | 18 (4.60) | 9 (8.57) |
| OR | 0.59 (0.43- 0.79) | 1 | 0.63 (0.37- 1.10) | 1.23 (0.59- 2.58) |
| aOR | 0.60 (0.43- 0.83) | 1 | 0.46 (0.25- 0.83) | 0.66 (0.28- 1.55) |
| Small for gestational age^a^ |  |  |  |  |
| n/N (%) | 267 (4.61) | 22 (2.76) | 119 (30.43) | 22 (20.95) |
| OR | 1.70 (1.09- 2.64) | 1 | 15.39 (9.56- 24.76) | 9.32 (4.95- 17.55) |
| aOR | 1.33 (0.81- 2.17) | 1 | 14.18 (8.27- 24.28) | 9.02 (4.24- 19.21) |
| Large for gestational age^a^ |  |  |  |  |
| n/N (%) | 775 (13.39) | 184 (23.11) | 42 (10.74) | 28 (26.66) |
| OR | 0.51 (0.42-0.61) | 1 | 0.40 (0.27-0.57) | 1.20 (0.76-1.92) |
| aOR | 0.54 (0.44-0.66) | 1 | 0.29 (0.19-0.44) | 0.78 (0.46-1.33) |
| Low Apgar score^b^ |  |  |  |  |
| n/N (%) | 216 (3.73) | 31 (3.89) | 53 (13.55) | 16 (15.24) |
| OR | 0.95 (0.65- 1.40) | 1 | 3.87 (2.44- 6.13) | 4.43 (2.33- 8.43) |
| aOR | 0.76 (0.42- 1.37) | 1 | 1.39 (0.64- 2.97) | 2.10 (0.71- 6.16) |
| Severe adverse neonatal outcomes^b^ |  |  |  |  |
| n/N (%) | 538 (9.30) | 94 (11.81) | 106 (27.11) | 30 (28.57) |
| OR | 0.76 (0.60- 0.96) | 1 | 2.77 (2.03- 3.78) | 2.98 (1.85- 4.80) |
| aOR | 0.71 (0.50- 1.00) | 1 | 1.15 (0.71- 1.88) | 1.49 (0.71- 3.14) |

Low apgar score: 1-min apgar<7 or 5-min apgar score<7.

Severe adverse neonatal outcomes: at least one of including stillbirth, resuscitation failure in delivery room, or admission to NICU.

^a^adjusted gravidity (2, 3, >3), parity (2, > 2), body mass index, abortion history (yes/no), interpregnancy interval and maternal age .

^b^adjusted gravidity (2, 3, >3), parity (2, > 2), body mass index, abortion history (yes/no), mode of the current delivery (vaginal/cesarean), interpregnancy interval, gestational age, birth weight and maternal age.
